# Comprehensive Analysis of KCNJ14 Potassium Channel as a Biomarker for Cancer Progression and Development

**DOI:** 10.3390/ijms24032049

**Published:** 2023-01-20

**Authors:** Glowi Alasiri

**Affiliations:** Department of Biochemistry, College of Medicine, Al Imam Mohammad Ibn Saud Islamic University (IMSIU), Riyadh 13317, Saudi Arabia; gaalasiri@imamu.edu.sa

**Keywords:** KCNJ14, pan-cancer analysis, K^+^ channel, cancer therapy, RNA modification

## Abstract

Cancer is a global epidemic that has affected millions of lives. Discovering novel cancer targets is widely viewed as a key step in developing more effective therapies for cancer and other fatal illnesses. More recently, potassium (K^+^) channels have been studied as a potential biological target for the creation of cancer treatments. Potassium Inwardly Rectifying Channel Subfamily J Member 14 (KCNJ14) is one of the cancer genome’s least investigated genes. This study conducted a comprehensive examination of the relationships between KCNJ14 gene expression analysis, survival, RNA modification, immunotherapy participation, and cancer stemness using several databases. KCNJ14 was shown to be dysregulated in a variety of cancers, including lung, intestinal, head and neck, oesophageal, and stomach. Additionally, KCNJ14 was shown to be linked to RNA and DNA stemness in 18 and 15 different tumour types, respectively. Moreover, KCNJ14 was discovered to be positively linked with immunological checkpoints and suppressor cells and to have a negative immunophenoscore (IPS). KCNJ14 was linked to tumour mutation burden (TMB), microsatellite instability (MSI), neoantigen (NEO), and programmed death ligand 1 (PD-L1); all four are potential targets for immunotherapies. In addition, a favourable relationship between genomic-instability markers such as heterozygosity (LOH), homologous recombination deficiency (HRD), and mutant-allele tumour heterogeneity (MATH) was demonstrated with KCNJ14. Based on these novel findings, KCNJ14 may be a useful independent prognostic biomarker for a range of cancers.

## 1. Introduction

Millions of individuals all over the world are burdened with cancer. Estimated to reach 28.4 million cases in 2040, up 47% from 2020, cancer is the second leading cause of mortality worldwide after cardiovascular disease, accounting for 10.0 million fatalities (9.9 million excluding non-melanoma skin cancer) in 2020 [1]. The five most common cancers in females are breast (24.5%), colorectal (9.4%), lung (8.4%), cervix (6.5%), and thyroid (4.9%). Meanwhile, lung (14.3%), prostate (14.1%), colorectal (10.6%), stomach (7.1%), and liver (6.3%) are the most prevalent form of malignancies among males [1].

The search for new therapeutic targets in cancer research has occupied a significant portion of the budget and time devoted to the disease. It is widely believed that the discovery of new targets for cancer will lead to the creation of effective new treatments for the deadly disease. Given their prevalence in cancer biology, their status as surface-expressed proteins, and the abundance of pharmacological techniques available to alter them, ion transport molecules have been frequently recommended as possible targets for the creation of new therapeutic treatments [2]. In this regard, K^+^ channels have gained attention as a promising biological target for the development of anti-cancer therapies in recent years [3]. K^+^ channels have been linked to cancer because they play a role in the pathways that drive cancer growth [4]. In biological membranes, K^+^ channels—complex proteins that form selective pores for K^+^ conduction—play a crucial role in K^+^ homeostasis, cell volume regulation, resting-membrane-potential establishment, the release of neurotransmitters, and the regulation of neuronal and skeletal muscle excitability [5].

Inward rectifying potassium channels (Kir) are ion channels that transport K^+^ ions into cells more efficiently than out of them [6]. Each Kir consists of four subunits, each of which has two transmembrane segments that surround a single pore loop (out of the cell) (Kuang, 2015) [7]. Among mammals, the Kir family is represented by 15 genes, divided into 7 subfamilies (Kir1.x to Kir7.x) [8]. Voltage-dependent blockage of Kir channels by magnesium (Mg^2+^) and polyamine controls their gating [8]. Numerous studies have reported dysregulated Kir family expression in human cancer. They have been linked to small-cell lung cancer, brain tumour, pancreatic ductal adenocarcinoma, breast carcinomas, and adrenal aldosterone-producing adenomas [4]. Additionally, the Kir family were reported to be involved in mechanisms of cancer development and progression, such as the cell cycle, reactive oxidative species, and metastasis [4].

KCNJ14 is one of the least well-studied K^+^ channels. KCNJ14 is located on chromosome 19q13, and the encoded protein has been linked to a variety of biological activities, including heart rate modulation, neurotransmitter release, epithelial electrolyte transport, and immunological regulation [9]. However, in relation to cancer, KCNJ14 has been reported as a biomarker for colorectal cancer (CRC) only [10].

In this study, KCNJ14 pan-cancer analysis was conducted to explore its association with cancer progression and development. In addition, KCNJ14’s role in immunotherapy was examined, as well as its involvement in cancer epigenetics and cancer stemness. This work identifies KCNJ14 as a possible prognostic biomarker for several cancer types.

## 2. Results

### 2.1. Expression and Pathological Analysis of KCNJ14

**Figure 1:**
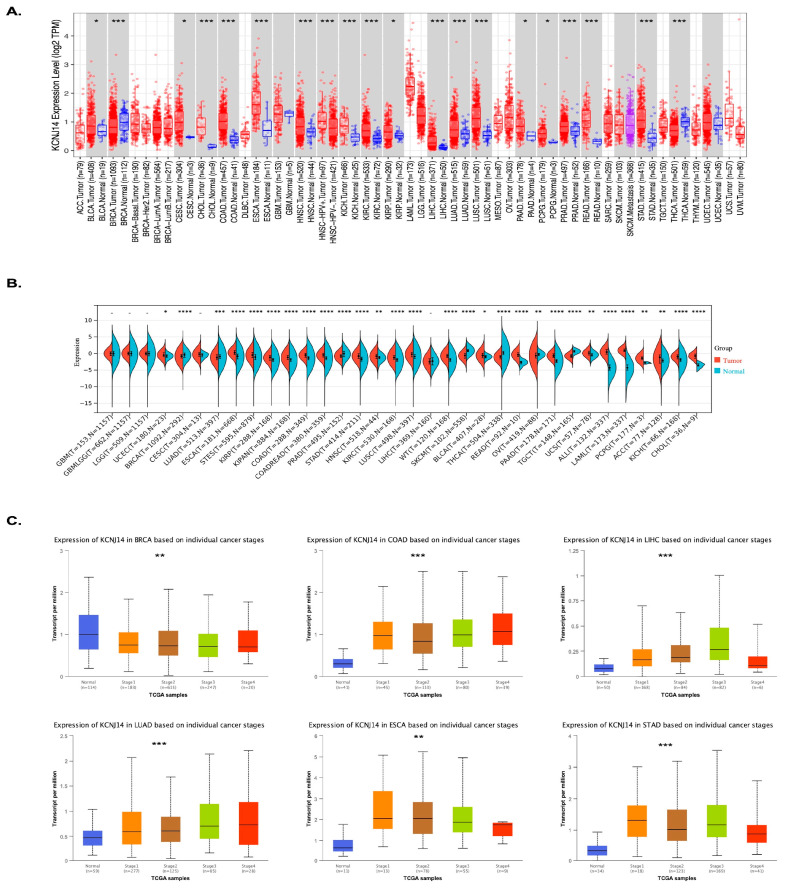
Tissue mRNA expression and pathological stages of KCNJ14. (**A**,**B**) represent tissue pan-cancer analysis of KCNJ14 mRNA expression using TIMER.2 and Sangerbox databases. * *p* < 0.05; ** *p* < 0.01; *** *p* < 0.001; **** *p* < 0.0001. (**C**) represents pathological stages’ differential expression of KCNJ14 in BRCA, COAD, LIHC, LUAD, ESCA, and STAD. The analyses were conducted using UALCAN database. ** *p* < 0.01; *** *p* < 0.001.

The role of KCNJ14 was previously examined in CRC, showing KCNJ14 as a prognostic biomarker in CRC [10]. In this work, KCNJ14 expression was examined across 37 types of cancers using the TIMER.2 database and Sangerbox database (Figure 1A,B). The data showed the upregulation of KCNJ14 in 22 tumour types, including uterine corpus endometrial carcinoma (UCEC, *p* = 0.05), oesophageal carcinoma (ESCA, *p* = 8.8 × 10^−36^), stomach and oesophageal carcinoma (STES, *p* = 1.9 × 10^−24^), kidney renal papillary cell carcinoma (KIRP, *p* = 1.1 × 10^−12^), pan-kidney cohort (KICH + KIRC + KIRP) (KIPAN, *p* = 3.2 × 10^−22^), colon adenocarcinoma (COAD, *p* = 3.0 × 10^−30^), colon adenocarcinoma/rectum adenocarcinoma oesophageal carcinoma (COADREAD, *p* = 1.2 × 10^−38^), stomach adenocarcinoma (STAD, *p* = 6.4 × 10^−24^), head and neck squamous cell carcinoma (HNSC, *p* = 1.6 × 10^−3^), kidney renal clear cell carcinoma (KIRC, *p* = 2.1 × 10^−22^), lung squamous cell carcinoma (LUSC, *p* = 2.7 × 10^−20^), high-risk Wilms tumour (WT, *p* = 2.7 × 10^−22^), bladder urothelial carcinoma (BLCA, *p* = 0.02), rectum adenocarcinoma (READ, *p* = 9.3 × 10^−7^), pancreatic adenocarcinoma (PAAD, *p* = 3.0 × 10^−39^), uterine carcinosarcoma (UCS, *p* = 1.5 × 10^−3^), acute lymphoblastic leukaemia (ALL, *p* = 7.0 × 10^−63^), acute myeloid leukaemia (LAML, *p* = 1.8 × 10^−76^), pheochromocytoma and paraganglioma (PCPG, *p* = 8.8 × 10^−3^), adrenocortical carcinoma (ACC, *p* = 1.0 × 10^−3^), kidney chromophobe (KICH, *p* = 1.3 × 10^−11^), cholangiocarcinoma (CHOL, *p* = 4.6 × 10^−6^), and LIHC. On the other hand, KCNJ14 was significantly downregulated in seven tumours, such as BRCA (*p* = 1.4 × 10^−7^), lung adenocarcinoma (LUAD, *p* = 2.7 × 10^−4^), prostate adenocarcinoma (PRAD, *p* = 2.0 × 10^−12^), skin cutaneous melanoma (SKCM, *p* = 1.0 × 10^−38^), thyroid carcinoma (THCA, *p* = 4.7 × 10^−86^), ovarian serous cystadenocarcinoma (OV, *p* = 1.0× 10^−3^), and testicular germ cell tumours (TGCT, *p* = 2.2 × 10^−42^). Interestingly, LUAD showed the opposite expression using the TIMER.2 database where it was upregulated. Therefore, KCNJ14 expression level in LUAD was further investigated using the UALCAN database, and the data showed it was upregulated (*p* = 7.36140004509167 × 10^−9^). 

Additionally, the pathological stages’ expression analysis of KCNJ14 was conducted via the UALCAN database (Figure 1C). Six types of tumours considered from the highest in incidence were selected, including BRCA, COAD, LIHC, LUAD, ESAD, and STAD [1]. The results demonstrated that KCNJ14 was downregulated with cancer progression in BRCA; however, KCNJ14 showed higher expression in correlation with the pathological stages of COAD, LIHC, LUAD, ESAD, and STAD (Table S1).

### 2.2. Survival Analysis

**Figure 2:**
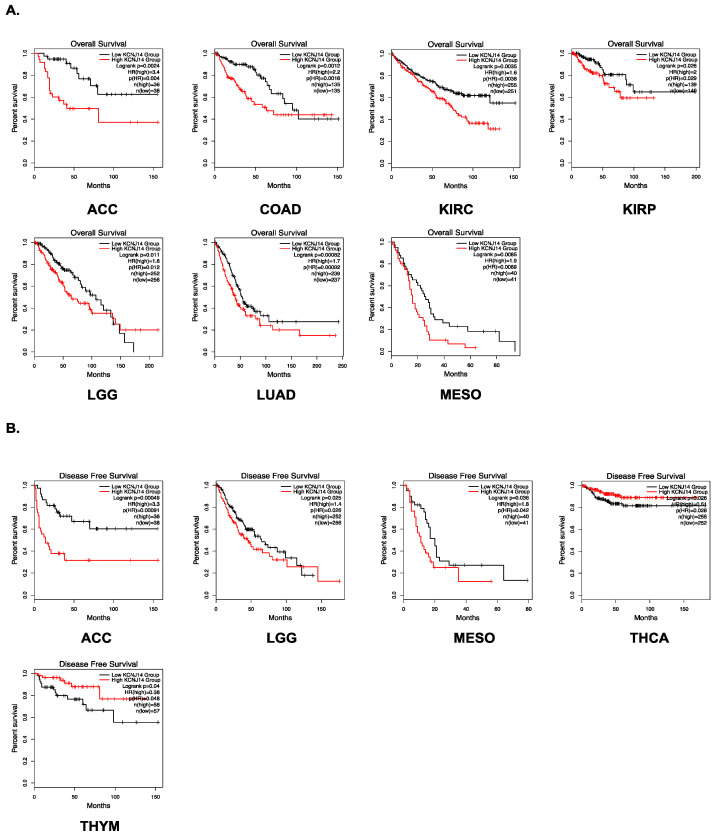
Survival level analysis of KCNJ14 in different tumour tissues. (**A**) represents overall survival map and Kaplan–Meier plot, whereas (**B**) represents the progression-free survival map and Kaplan–Meier plot. GEPIA2 tool was used to extract these data.

KCNJ14 overexpression has been demonstrated to induce proliferation in CRC [10]. These data further substantiate its oncogenic role in cancer. KCNJ14 has a poor prognosis in terms of overall survival (OS) in ACC, COAD, KIRC, KIRP, LGG, LUAD, MESO, and LIHC, as shown by survival analysis utilising the GEPIA.2 and Kaplan–Meier plotter databases (Figure 2A and Figure S1). Furthermore, poor (disease-free survival) DFS was associated with high KCNJ14 expression in ACC, LGG, MESO, THCA, and THYM (Figure 2B). KCNJ14, on the other hand, was an excellent prognostic indication for BRCA (Figure S1).

### 2.3. Genetic Alteration

**Figure 3:**
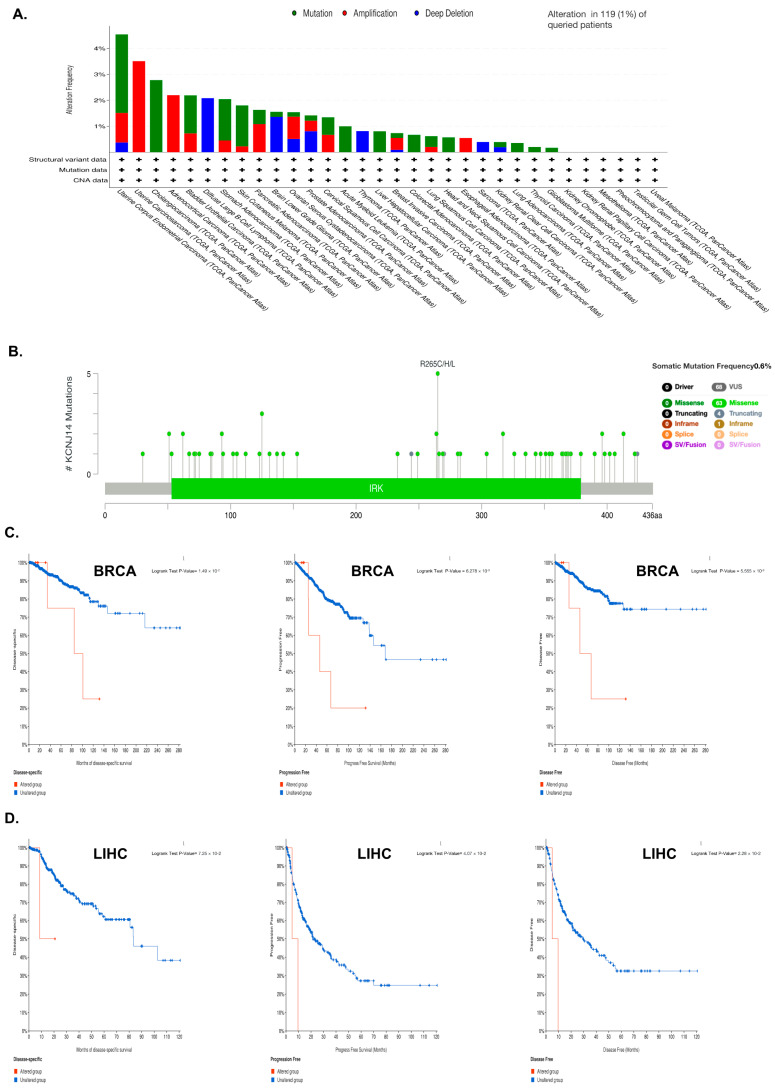
Genetic alteration of KCNJ14 in multiple cancer tissues. (**A**) represents mutation types and frequency in all TCGA tumours, whereas (**B**) represents the mutation site for all types of tumours. (**C**,**D**) represent the effect of mutation on patient survival in BRCA and LIHC.

KCNJ14 mutations were studied by utilising the cBioPortal database for information on their prevalence, mutation location, and prognostic impact in cancer (Figure 3). Data analysis revealed that KCNJ14 mutated in 1% of 26 types of cancers, with amplification being the main kind of mutation in 12.84 % of these cases (Figure 3A). Moreover, a diagram of KCNJ14 mutation sites revealed that the frequency of somatic mutation was 0.6%, with R265C/H/L being the most changed amino acid sequence (Figure 3B). Furthermore, the impact of these mutations on specific types of cancer, such as BRCA and LIHC, was investigated (Figure 3C and Figure S2). Both BRCA and LIHC patients with a KCNJ14 mutation had poor disease-specific survival (DSS), PFS, and DFS, but not OS.

### 2.4. KCNJ14 and RNA Methylation Modification Markers

**Figure 4:**
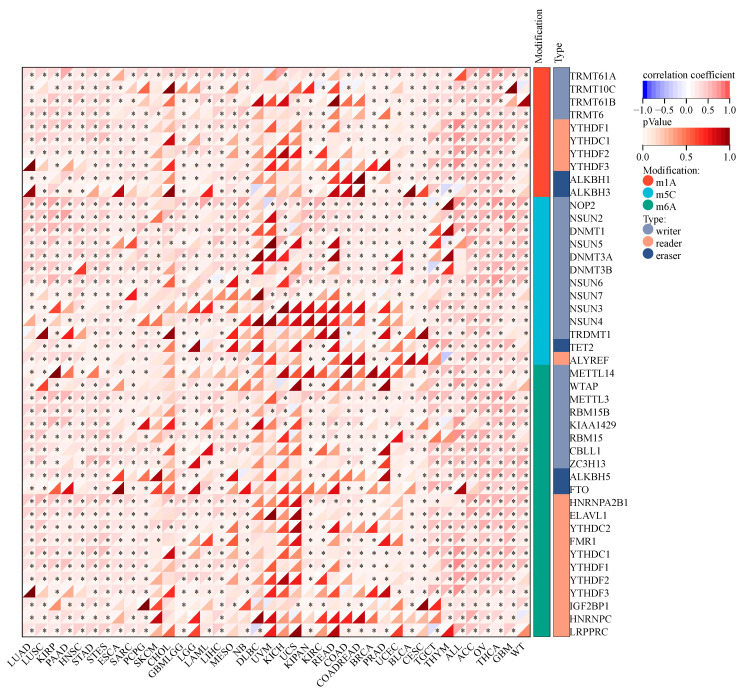
Correlation of RNA modifications (m1A, m5C, and m6A) and KCNJ14 in different types of cancers (* *p* < 0.05).

Alterations in RNA methylation have been linked to the initiation and progression of cancer and the response of cancer to therapy. This study examined how KCNJ14 is linked to RNA methylation alterations (Figure 4). According to the findings, KCNJ14 had a strong positive correlation with most genes involved in modifying RNA methylation. In contrast, several genes showed no significant correlation with cancer tissues, such as DLBC, UVM, KICH, and UCS. These results provide the first direct proof that KCNJ14 is involved in regulatory networks.

### 2.5. Genomic-Instability Parameters

**Figure 5:**
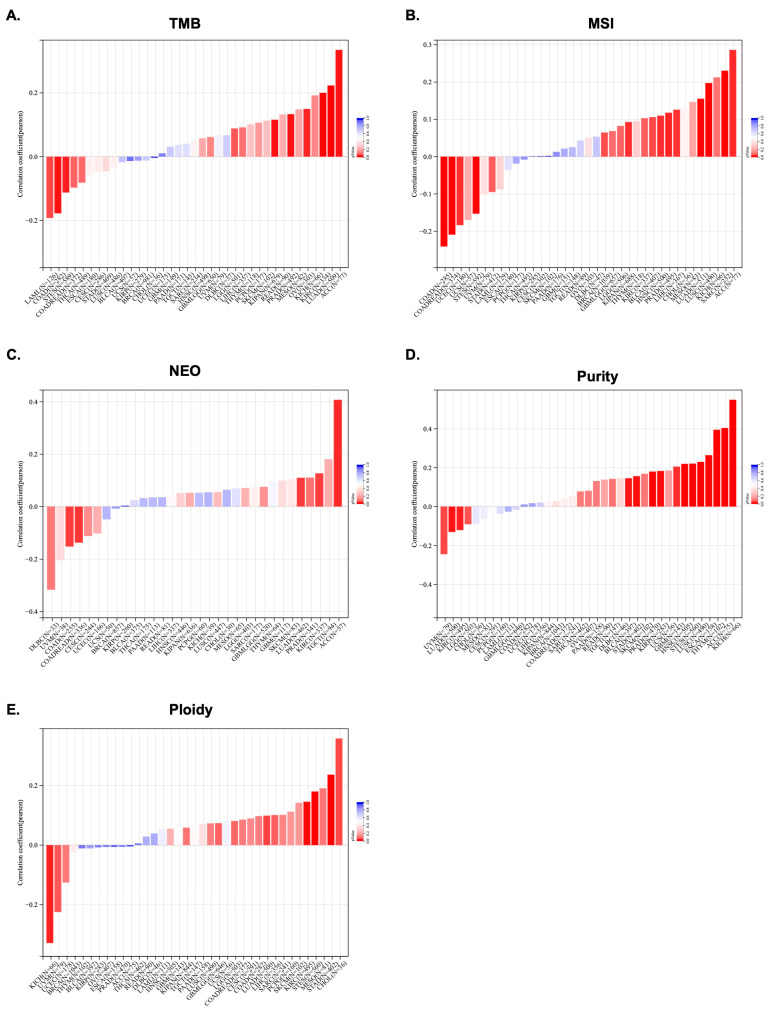
Pan-cancer analysis of KCNJ14 in correlation with tumour heterogenicity markers. (**A**) represents TMB; (**B**), MSI; (**C**), NEO; (**D**), purity; and (**E**), ploidy. Dark-red bars indicate “significant” while blue bars indicate “insignificant”.

**Figure 6:**
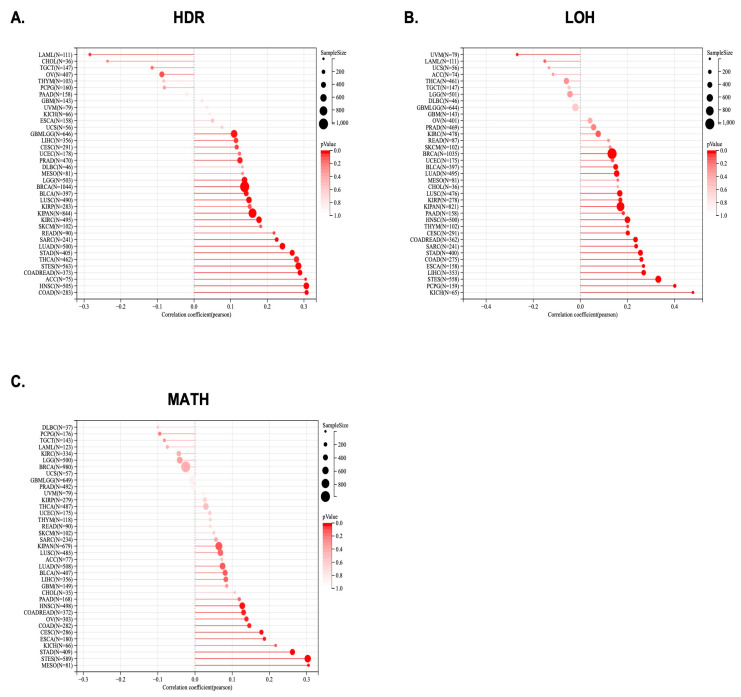
LOH, HRD, and MATH genomic-instability markers and KCNJ14 gene expression. (**A**) represents LOH; (**B**), HRD; and (**C**), MATH. Lollipop charts are displayed where the size of the dots represents the sample size, and the colour represents the *p*-value.

To further investigate KCNJ14’s role as a potential therapeutic target, this work explored the correlation of genomic-instability markers such as TMB, MSI, NEO, purity, ploidy, HRD, LOH, and MATH (Figure 5 and Figure 6).

In terms of TMB, the data showed a significant correlation in 10 tumours, among which a significant positive correlation was found in 7 tumours, such as LGG, LUAD, KIPAN, PRAD, KIRC, OV, and ACC, and a significant negative correlation was found in 3 tumours, namely COAD, LAML, and STES (Figure 5A). Additionally, MSI positively correlated with KCNJ14 in 10 tumours, including LUAD, BRCA, SARC, KIPAN, PRAD, HNSC, LUSC, LIHC, BLCA, and ACC, but negatively correlated with KCNJ14 in 4 tumours, namely COAD, COADREAD, UCEC, and STES (Figure 5B). Furthermore, KCNJ14 associated with NEO in five tumours, including a positive correlation with LUAD, KIRC, and ACC and a negative correlation with COAD and COADREAD (Figure 5C).

Tumour purity is significantly related to the clinical characteristics, genome expression, and biological characteristics of tumour patients. The influence of tumour purity should be fully considered when analysing tumour samples during the research process. Therefore, KCNJ14’s correlation with purity was investigated, and the result showed a significant correlation in 16 tumours, among which a significant positive correlation was found in 12 tumours, such as GBM, ESCA, STES, KIRP, STAD, PRAD, HNSC, LUSC, THYM, BLCA, ACC, and KICH, and a significant negative correlation was found in 4 tumours, such as LUAD, KIRC, and UVM (Figure 5D). Moreover, polyploidy is a hallmark of cancer and is closely associated with chromosomal instability involved in cancer development and in estimating tumour purity. Ploidy is beneficial for the study of cancer genome evolution and intratumor heterogeneity. In this work, a significant correlation between KCNJ14 and ploidy level was observed in seven tumours, of which five tumours demonstrated a significantly-positive correlation, such as LUAD, STES, STAD, KIRC, and CHOL, whereas two tumours showed a negative correlation, namely UVM and KICH (Figure 5E).

Significant correlations between KCNJ14 with LOH and HRD were found in several tumours, including GBMLGG, LGG, CESC, LUAD, COAD, COADREAD, BRCA, STES, SARC, KIPAN, STAD, PRAD, HNSC, KIRC, LUSC, LIHC, THCA, READ, BLCA, ACC, and LAML (Figure 6A,B). Moreover, KCNJ14 was associated positively with MATH in nine tumours, including CESC, COAD, COADREAD, ESCA, STES, STAD, HNSC, MESO, and OV (Figure 6C).

### 2.6. KCNJ14 Showed a Positive Association with Tumour Stemness

**Figure 7:**
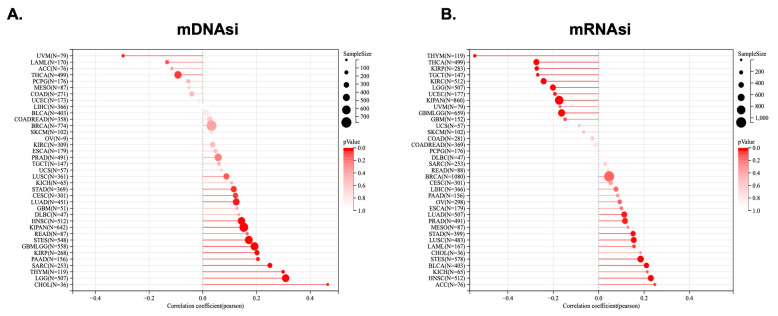
Association of KCNJ14 with cancer stemness. (**A**) represents mDNAsi, and (**B**) represents mRNAsi. Lollipop charts are displayed where the size of the dots represents the sample size, and the colour represents the *p*-value.

Tumour stemness was analysed in correlation with KCNJ14 using parameters such as DNAss and RNAss (Figure 7). The mDNAsi results showed a significant correlation with 15 tumours, among which a significant positive correlation was found in 13 tumours, such as GBMLGG, LGG, CESC, LUAD, STES, SARC, KIRP, KIPAN, STAD, HNSC, THYM, PAAD, and CHOL, whereas 2 tumours showed a significant negative correlation, namely THCA and UVM (Figure 7A). Additionally, mRNAsi demonstrated a significant correlation in 18 tumours, of which 9 tumours positively correlated with KCNJ14, including LUAD, LAML, STES, STAD, PRAD, HNSC, LUSC, BLCA, and ACC, and 9 tumours showed a significant negatively correlation with KCNJ14, namely GBML5G9, LGG, KIRP, KIPAN, UCEC, KIRC, THYM, THCA, and TGCT (Figure 7B).

### 2.7. Correlation of KCNJ14 with Immune Infiltration Cells

**Figure 8:**
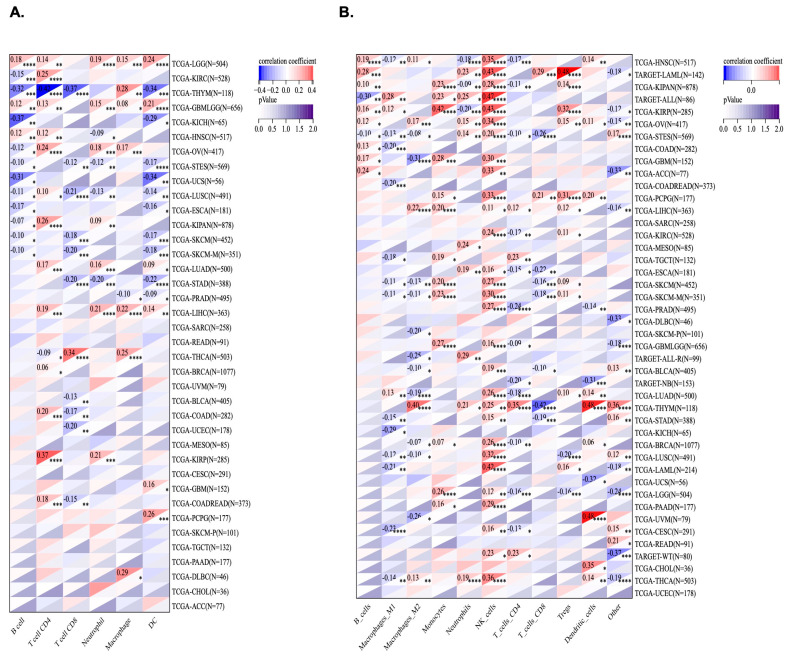
KCNJ14 and TIMER/QUANTISEQ-based investigation of correlations with tumour immune cell infiltration. (**A**) represents TIMER in 38 distinct cancers, and (**B**) represents QUANTISEQ for 42 types of cancers (* *p* < 0.05; ** *p* < 0.01; *** *p* < 0.001; **** *p* < 0.0001).

Using TIMER and the QUANTISEQ algorithm, KCNJ14’s correlation with immune infiltration cells was investigated (Figure 8). Out of 38 tumours, 28 tumours were significantly correlated with KCNJ14 in the TIMER database. LGG and GMBLGG tissues were associated positively with B cells, T-cell CD4, neutrophile, macrophage, and dendric cells. Moreover, T-cell CD4 correlated positively with COAD, COADREAD, LUAD, LUSC, BRCA, LIHC etc. Conversely, T-cell CD8 correlated negatively with several cancers, including COAD, COADREAD, LUSC, STES, and STAD (Figure 8A). QUANTISEQ algorithm analysis revealed 42 tumours correlated significantly with the expression of KCNJ14 of which 26 types of tumours showed a positive correlation with natural killer cells (Figure 8B).

### 2.8. KCNJ14 Correlates with Immunoregulatory Genes and Immune Checkpoint Genes

**Figure 9:**
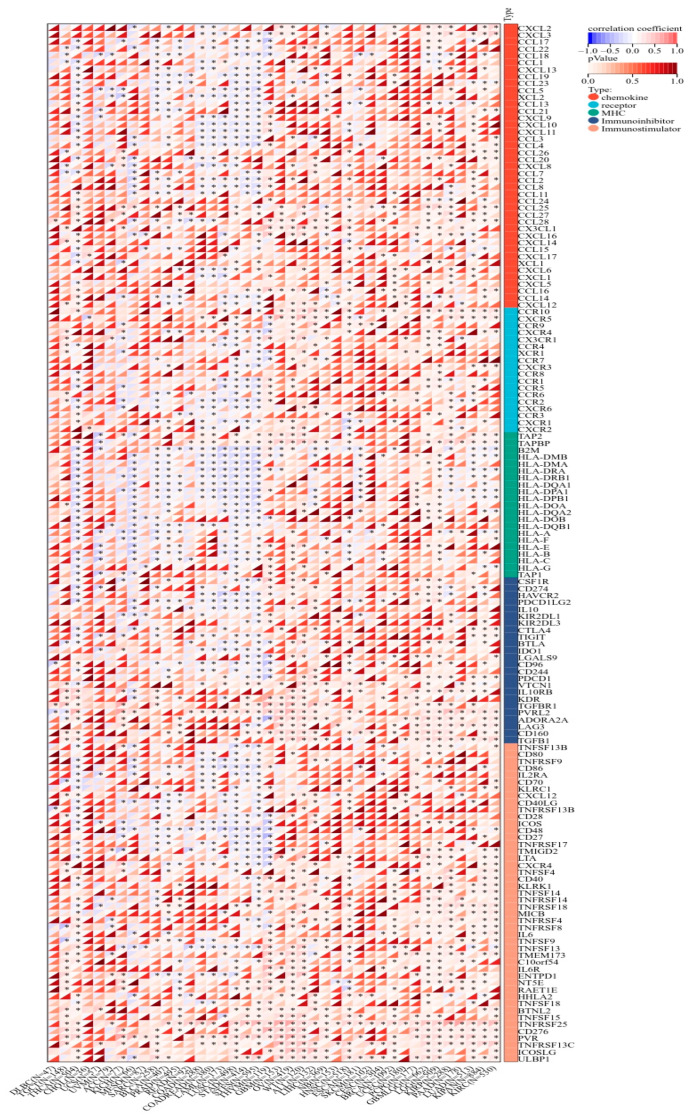
Cancer-wide association between KCNJ14 and genes for chemokines and their receptors. KCNJ14 expression was found to be highly associated with chemokine receptors belonging to the CXC and CC families, major histocompatibility complex class I and class II molecules, and immunoinhibitors and immunostimulators (* *p* < 0.05).

**Figure 10:**
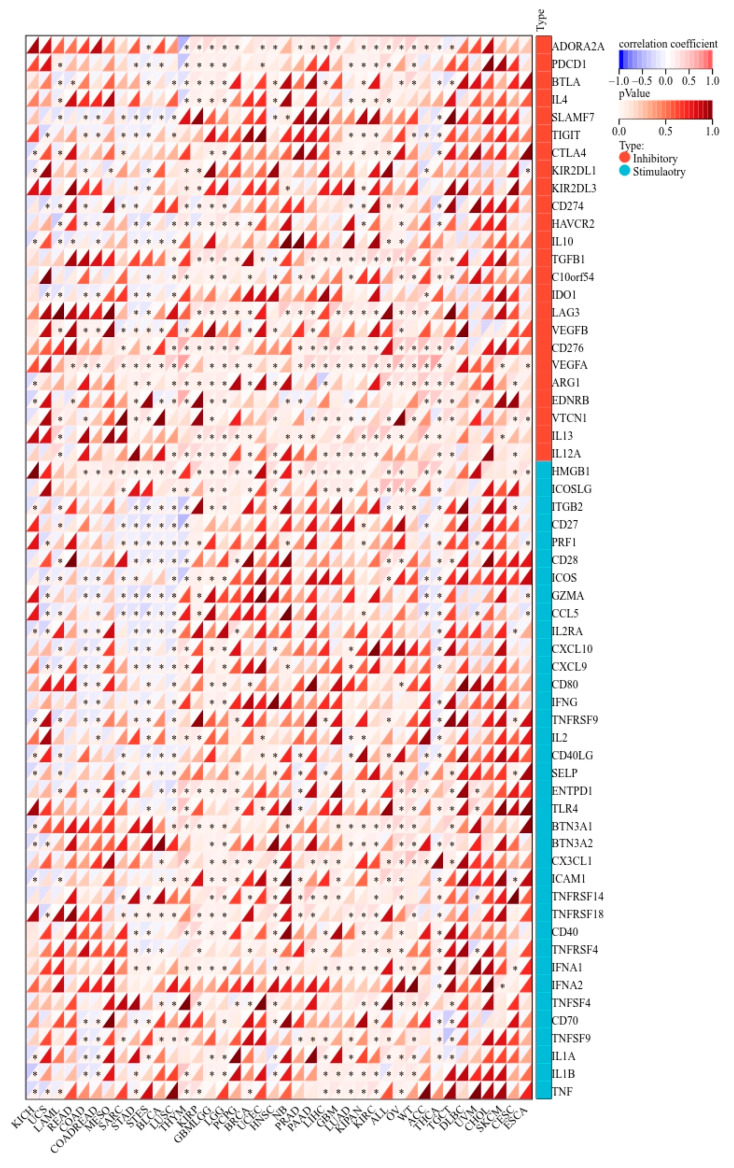
Association between KCNJ14 with genes inhibiting and stimulating immunological checkpoints in cancers (* *p* < 0.05).

KCNJ14 demonstrated a strong correlation with chemokines, receptors, and immunostimulatory and immunoinhibitory markers (Figure 9). Most cancer tissues were associated positively except for THYM, STES, STAD, LUSC, LAML, COADREAD, and COAD, which showed a negative correlation with several chemokine chemokines, receptors, and immunostimulatory and immunoinhibitory genes. Additionally, KCNJ14 demonstrated a correlation with inhibitory and stimulatory immune checkpoints with respect to tumours such as THYM, STES, STAD, LUSC, LAML, COADREAD, and COAD (Figure 10). Furthermore, multiple malignancies exhibited substantial connections between KCNJ14 and immune checkpoint inhibitory genes such as VEGFA, CD274(PD-L1), CTLA4, etc. In addition, KCNJ14 was linked significantly with several stimulatory immune checkpoints, including IFNA1, TNFRSF14, TNFRSF18, etc.

### 2.9. Using Immunophenoscore (IPS) to Study KCNJ14’s Association with Immune Infiltrations Cell

**Figure 11:**
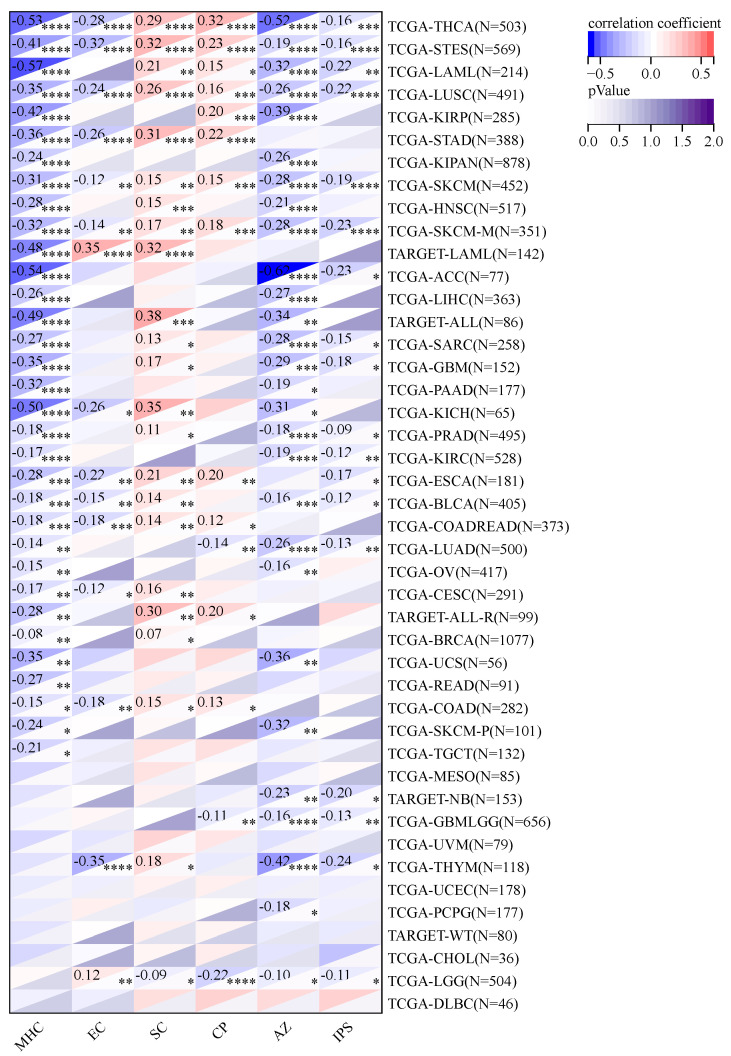
Evaluation of KCNJ14 association with tumour immune system using IPS. Immunophenoscore was adversely linked with KCNJ14 expression. In the figure, CP refers to checkpoints; EC, effector cells; SC, suppressor cells; IPS, immunophenoscore; and MHC, major histocompatibility antigen-processing pathway (* *p* < 0.05; ** *p* < 0.01; *** *p* < 0.001; **** *p* < 0.0001).

In terms of checkpoints and suppressor cells, KCNJ14 showed a positive correlation with multiple types of tumours, except LGG, GMBLGG, and LUAD (Figure 11). Furthermore, KCNJ14 demonstrated a negative correlation with IPS in 18 types of cancers including THCA, STES, LAML, LSC, SKCM, ACC, PRAD, LUAD, etc.

## 3. Discussion

Cancer is a set of disorders that originate from alterations in biochemical pathways that, in turn, promote cell proliferation [11]. Ion channels are historically known to regulate many cellular functions, including electrical-signal generation and transmission, secretion, and contraction through the regulation of ionic gradients [12]. The K^+^ ion channel was demonstrated previously in several cancers’ progression and development [13]. This study examines the role of KCNJ14 as a potential prognostic biomarker for a variety of malignancies, including (but not limited to) BRCA, LUAD, ESAD, STAD, LIHC, and GMBLGG.

Previous research indicated that increased levels of KCNJ14 expression in CRC tissues and cell lines were correlated with shorter median survival rates for CRC patients [10]. Moreover, KCNJ2, another Kir channel family, was found upregulated in small-cell lung cancer with a significant role in clinical stages and treatment response [14]. The results of this work consistently revealed that the overexpression of KCNJ14 in ACC, KIRC, KIRP, LGG, LUAD, MESO, and LIHC was related to reduced survival rates. Furthermore, it was observed that KCNJ14 was associated with PFS, and its overexpression was coupled with a worse prognosis. Additionally, KCNJ14 was found to be associated with cancer progression as its expression increased with pathological stages. Interestingly, BRCA tissue revealed contrasting findings whereby it indicated KCNJ14 as a possible favourable prognostic marker due to its overexpression in normal tissue and augmentation of survival rate.

Cancer stem cells (CSCs) behave as their malignant equivalents and can persist even after therapy [15]. Increasing our knowledge of the CSCs’ microenvironment is key to identifying new therapeutic solutions. Despite their minimal stemness, MSCs are a crucial part of the stem cell niche and tumour microenvironment [15]. It was previously reported that KCNJ3 overexpression contributed to cancer cells’ self-renewal and stemness [16]. In this analysis, KCNJ14 was found to have a significant positive connection with mDNAsi and mRNAsi in a total of 13 and 9 tumours, respectively. These findings are the first to suggest a link between KCNJ14 and cancer stemness.

Many biological processes, such as development, ageing, and disease (including cancer), are regulated by epigenetic modifications [17]. Phenotypic alterations can occur due to heritable epigenetic modifications, which do not involve changes to the nucleic acid sequence [18]. The modification of RNA can occur on any of the four nucleotides (A, U, C, or G). Moreover, there are over 170 RNA modifications, such as m5C, m3C, m7G, pseudouracil, and Nm modification [19]. Additionally, tumour development, progression, metastasis, and immunological modulation are all profoundly influenced by RNA modification [20]. In this study, KCNJ14 expression was found to have a substantial positive connection with RNA-modification-related genes (m5C, m3C, and m7G) for a wide range of cancers. Therefore, this finding provides a novel insight into the role of KCNJ14 in RNA modification.

Tumour-infiltrating immune cells (TICs) can alter immunological processes associated with tumour growth, metastasis, and medication resistance [21]. TICs are functionally split into two categories—(i) tumour-cell-growth inhibition, including CD8+ T, Th1 CD4+ T, Th9 CD4+ T, plasma, memory B, NK cells, and DCs; and (ii) tumour-cell-growth or immune-escape stimulation, including Treg, Breg, macrophage M2, and myeloid-derived suppressor cells (MDSCs) [21,22]. The fact that KCNJ14 primarily affects the infiltration of CD4+ and CD8+ cells was first reported in CRC, which is of importance because CD4+ T cells play a major role in controlling the cancer immune microenvironment [10]. This paper demonstrated that KCNJ14 correlates with various TICs, such as T-cell CD8 and T-cell CD4, thereby further suggesting the involvement of KCNJ14 in tumour microenvironment (TME) regulation which, in turn, is significant in tumorigenesis and the development of anti-tumour therapy. 

Furthermore, this study found a positive relation between KCNJ14 and immunotherapy indicators, such as TMB, MSI, NEO, purity, and ploidy, in a variety of cancers. Studies in humans have shown that CD274 (PD-L1) expression level is crucial to the success of immune checkpoint inhibitor (ICI) therapy in different cancers [23]. It has been hypothesised that patients with increased PD-L1 expression will respond in a better way to ICIs [24]. In this study, CD274 positively correlated with KCNJ14 in OV, ALL, KIPAN, LUAD, LGG, GMBLGG, and THYM, which suggests its significance as an immunotherapy target.

Copy number variations, or chromosomal instability, are among the most influential alterations in cancer development and progression [25]. In this work, KCNJ14’s relation to chromosomal instability was studied using several algorithms, such as HRD, LOH, and MATH, and the data obtained revealed a positive connection in many malignancies (COAD, PAAD, STAD, LIHC, BRCA, KIRC, etc.). The mutation effect of the K^+^ channel was previously discussed with regard to KCNJ5 [26]. In adrenal aldosterone-producing adenomas (APAs), KCNJ5’s gain-of-function mutation related to the progression of APAs [27]. Additionally, this study provided more evidence for the association of genomic instability with K^+^ channel dysregulation.

Although this work has explored the K^+^ channel role of KCNJ14 in cancer, it still has limitations with respect to its view. Possible mechanisms, such as signalling-pathway regulation, stemness maintenance, RNA modification, and TME regulation, have been anticipated only at the bioinformatics level and need further validation using wet-lab experiments. Furthermore, KCNJ14’s role in response to chemotherapy treatment and resistance needs to be further elucidated, as it has been demonstrated in several studies that the ion channel is important in developing drug resistance [28]. 

In conclusion, overexpression of the oncogene KCNK14 was shown to be associated with poor health outcomes, and its overexpression was found to occur in a wide variety of human malignancies. Immunosuppressive cell (MDSC) infiltration was shown to correlate significantly with KCNJ14 expression. Additionally, TMB and MSI were linked to KCNJ14 expression in a variety of human malignancies, which suggests that these findings may establish KCNJ14 as a robust predictive biomarker, a marker for patients’ response to immunotherapy, and a prospective target for cancer therapies. Lastly, the involvement of genomic instability with KCNJ14 was demonstrated, which implies that it may be a suitable candidate for therapeutic intervention.

## 4. Materials and Methods

### 4.1. Gene Expression Analysis

Tumour Immune Estimation Resource version 2 (TIMER2.0) (http://timer.cistrome.org, accessed on 25 October 2022) was used to analyse the mRNA expression level of KCNJ14 in several types of tumours [29]. Additionally, the Sangerbox database (http://www.sangerbox.com/home.html, accessed on 25 October 2022) was accessed to demonstrate KCNJ14 gene expression in additional tumour tissues where there was a lack of normal tissue in some types of cancers in the TIMER2.0 database [30]. Furthermore, KCNJ14 gene expression in pathological stages was investigated using the UALCAN database (http://ualcan.path.uab.edu, accessed on 25 October 2022), which carries out gene expression analysis using the TCGA database [31].

### 4.2. Survival Analysis

The correlation between KCNJ14 expression and overall survival and disease-free survival was analysed using the Survival Map Module of the Gene Expression Profiling Interactive Analysis.2 (GEPIA2.0) (http://gepia2.cancer-pku.cn/#index, accessed on 25 October 2022) database [32]. In addition, Kaplan–Meier Plotter Pan-Cancer mRNA was used to explore KCNJ14 gene expression in breast invasive carcinoma (BRCA) and liver hepatocellular carcinoma (LIHC) [33].

### 4.3. Genetic Alteration

The Cancer Genomics Dataset from the TCGA Pan-Atlas (cBioPortal) (http://cbioportal.org, accessed on 25 October 2022) was used in the analysis of genetic alterations in KCNJ14 [34]. The frequency of the mutant gene is indicated in the section on cancer types. The “cancer types of summary” module was used to collect data on the frequency of alterations, mutation types, and copy number alterations in all TCGA tumours. The “mutations” module was used to analyse the KCNJ14 protein structure diagram and locate the mutation’s precise location. Overall survival (OS), progression-free survival (PFS), and disease-free survival (DFS) comparisons between TCGA patients, with and without KCNJ14 mutations, were calculated using the module’s namesake in BRCA and LIHC only. Log-rank *p*-values were displayed using Kaplan–Meier graphs.

### 4.4. RNA Modification

Analysis of RNA methylation modifications, including N1-methyladenosine (m1A), 5 methylcytidine (m5C), and N6-methyladenosine (m6A), was performed using the Sangerbox database. Writers, readers, and erasers are the three types of genes associated with modification. KCNJ14 expression was analysed in connection to genes involved in RNA methylation alterations in a variety of tumour types.

### 4.5. Genomic Instability

Utilising the online data analysis tool Sangerbox, the connection between KCNJ14 expression and genomic instability in cancers was evaluated. SangerBox is a comprehensive and free online tool for analysing TCGA data, which was utilised to investigate the relationships between KCNJ14 gene expression and HRD, LOH, NEO, MATH, purity, ploidy, DNA stemness score (DNAss), and RNA stemness score (RNAss) in multiple cancers.

### 4.6. Immune Infiltration Analysis and Immune Checkpoints

As immune infiltrations, IPS and immune checkpoints are valuable indicators of the tumour’s microenvironment. The study used SangerBox, a free online tool for processing TCGA data. The level of immune cell infiltration into TCGA tissue was evaluated using the TIMER and expression data (ESTIMATE) methods. In the existing study, 47 immune checkpoint genes were tested for correlation with KCNJ14 expression, and the results were analysed using Spearman’s rank test.

### 4.7. Statistical Analysis

The Wilcoxon test was used to examine the differential expression analyses between tumours and normal tissues and was performed with TIMER. Log-rank tests for calculating hazard ratio (HR) and log-rank *p*-value within the Kaplan–Meier plotter were used for comparing survival curves. Spearman’s correlation was utilised to investigate the link between gene expression levels. The results were considered significant if the probability coefficient was less than 0.05.

## Data Availability

The data were collected from public databases including UALCAN (http://ualcan.path.uab.edu/analysisprot.html, accessed on 25 October 2022), TIMER (http://timer.cistrome.org/), Sangerbox (http://sangerbox.com), and GEPIA.2 (http://gepia2.cancer-pku.cn/#index, accessed on 25 October 2022).

## Supplementary Materials

The following supporting information can be downloaded at: https://www.mdpi.com/article/10.3390/ijms24032049/s1.
