# Supplementary material for: Comprehensive Analysis of KCNJ14 Potassium Channel as a Biomarker for Cancer Progression and Development
Source: Int J Mol Sci. 2023 Jan 20;24(3):2049. doi: 10.3390/ijms24032049 (PMC9916715; doi:10.3390/ijms24032049)
Supplement: Supplementary file 1 [file ijms-24-02049-s001.zip › Supplementary Figures Captions.pdf]

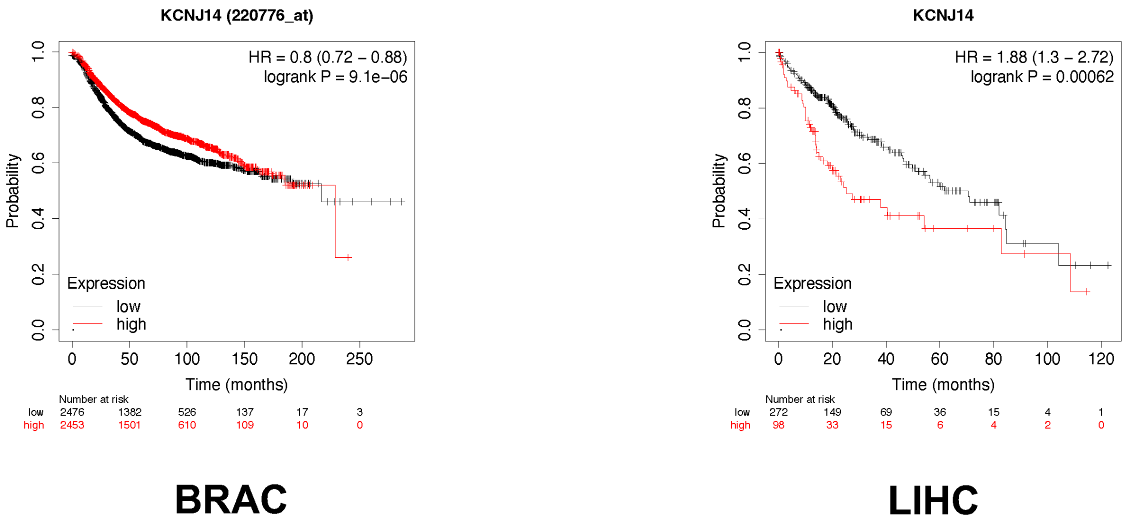

Figure S1. Survival level analysis of KCNJ14 in BRCA and LIHC.

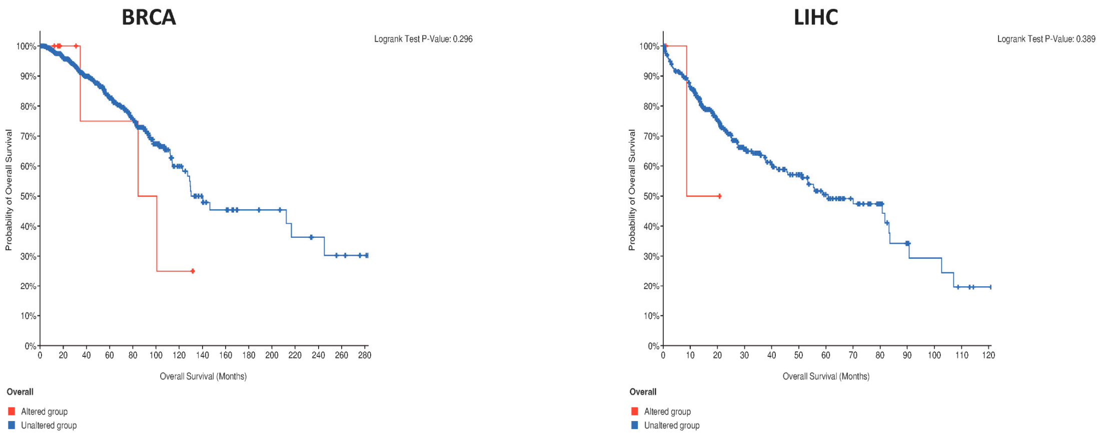

Figure S2. Represent the effect of mutation on patient overall survival in BRCA and LIHC.
