# Supplementary material for: Comprehensive Analysis of KCNJ14 Potassium Channel as a Biomarker for Cancer Progression and Development
Source: Int J Mol Sci. 2023 Jan 20;24(3):2049. doi: 10.3390/ijms24032049 (PMC9916715; doi:10.3390/ijms24032049)
Supplement: Supplementary file 1 [file ijms-24-02049-s001.zip › Table S1. Summary of KCNJ14 epression p- values in relation with stages of Six type of cancer Tissue.pdf]

Table S1. Summary of KCNJ14 epression p- values in relation with stages of Six type of cancer Tissue

Tissue	Stage 1 (S1)	Stage 2 (S2)	Stage 3 (S3)	Stage 4 (S4)
**BRCA**	P= 6.899100e-03	P= 4.625600e-02	P= 1.877770e-03	P= 4.498400e-02
**COAD**	P= 1.72990510804993e-11	P= <1e-12	P= 1.62447832963153e-12	P= 8.65019167406444e-12
**LIHC**	P= 1.63424829224823e-12	P= 1.09709999973617e-08	P= 9.6668228977137e-12	P= 2.041400e-01
**LUAD**	P= 4.03130000004026e-06	P= 7.53420000000338e-05	P= 1.55629999999407e-05	P= 1.165880e-02
**ESAD**	P= 5.420800e-03	P= 2.6120999999657e-06	P= 3.85529999999967e-05	P= 1.079520e-02
**STAD**	P= 2.995100e-04	P= 2.09943173956617e-12	P= 1.62447832963153e-12	P= 1.53410000003351e-06
