# Supplementary figures and images for: Comprehensive Analysis of KCNJ14 Potassium Channel as a Biomarker for Cancer Progression and Development
Source: Int J Mol Sci. 2023 Jan 20;24(3):2049. doi: 10.3390/ijms24032049 (PMC9916715; doi:10.3390/ijms24032049)

KCNJ14 (220776\_at)

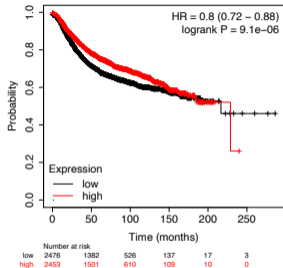

BRAC

KCNJ14

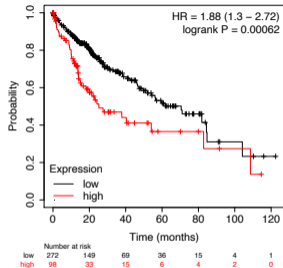

LIHC

Supplement: Supplementary file 1 [file ijms-24-02049-s001.zip › Figure S1.pdf]

## BRCA

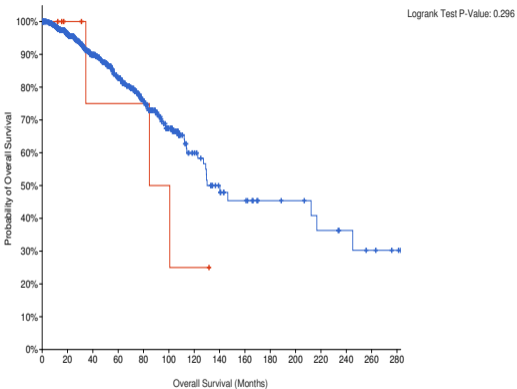

Overall

- Altered group
- Unaltered group

## LIHC

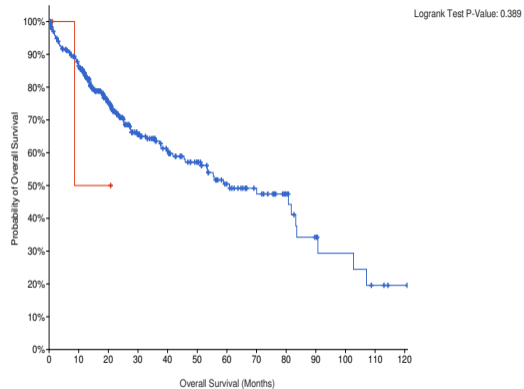

Overall

- Altered group
- Unaltered group

Supplement: Supplementary file 1 [file ijms-24-02049-s001.zip › Figure S2.pdf]
